# Supplementary material for: Longitudinal Study of DNA Methylation and Epigenetic Clocks Prior to and Following Test-Confirmed COVID-19 and mRNA Vaccination
Source: Front Genet. 2022 Jun 3;13:819749. doi: 10.3389/fgene.2022.819749 (PMC9203887; doi:10.3389/fgene.2022.819749)
Supplement: Supplementary file 3 [file DataSheet1.docx]

Longitudinal study of DNA methylation and epigenetic clocks prior to and following test-confirmed COVID-19 and mRNA vaccination

SUPPLEMENTARY MATERIALS

Alina PS Pang^1#^, Albert T. Higgins-Chen^2,3#^, Florence Comite^4,5^, Ioana Raica^4^, Christopher Arboleda^4^, Hannah Went^6^, Tavis Mendez^6^, Michael Schotsaert^7,8^, Varun Dwaraka^6^, Ryan Smith^6^, Morgan E. Levine^9^, Lishomwa Ndhlovu^1^, Michael J. Corley^1^*

^1^ Division of Infectious Diseases, Department of Medicine, Weill Cornell Medicine, New York, NY, USA

^2^Department of Psychiatry, Yale University School of Medicine, New Haven, CT, USA

^3^VA Connecticut Healthcare System, West Haven, CT, USA

^4^Comite Center for Precision Medicine & Health, New York, NY, USA

^5^Lenox Hill Hospital/Northwell, New York, NY, USA

^6^TruDiagnostic, Lexington, KY, USA

^7^Department of Microbiology, Icahn School of Medicine at Mount Sinai, New York, NY, USA

^8^Global Health and Emerging Pathogens Institute, Icahn School of Medicine at Mount Sinai, New York, NY, USA

^9^Department of Pathology, Yale University School of Medicine, New Haven, CT, USA

**#Authors Contributed Equally**

**Supplementary** **Figure S1.** Enrichr gene enrichment showing top biological processes and KEGG pathways for genes associated with differentially methylated loci.

**
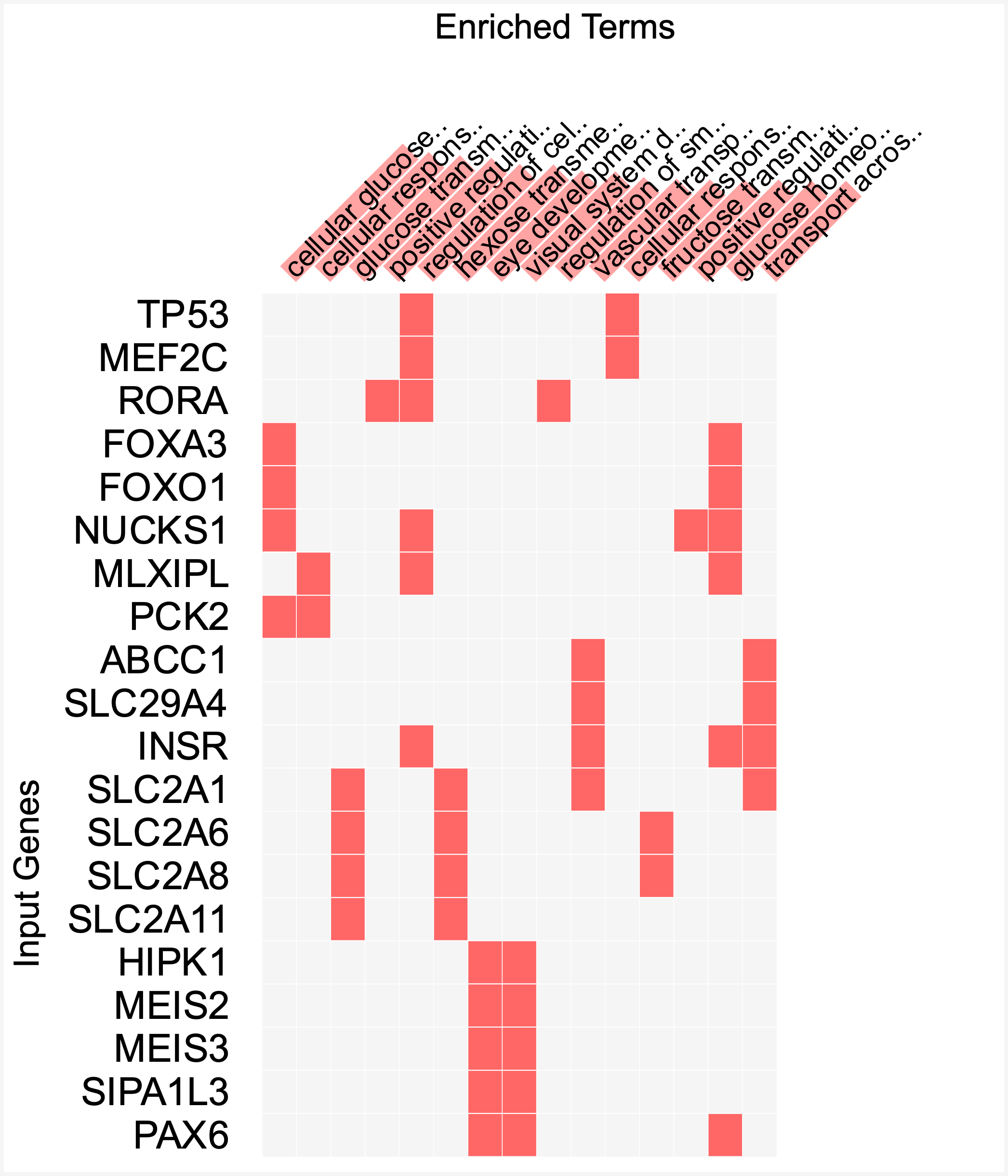

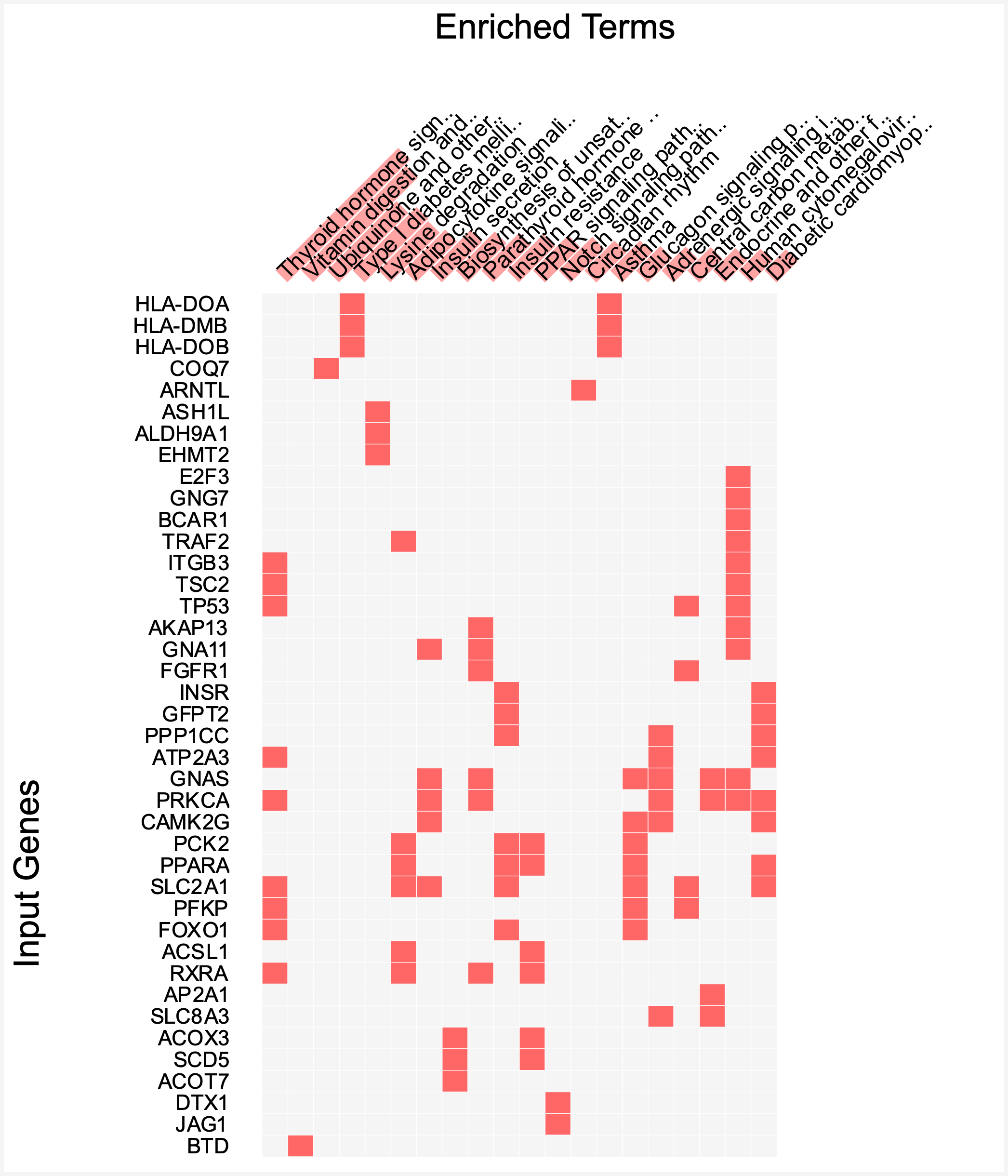
**

**Supplementary** **Figure S2.** Gene promoter transcription start site regulatory region of the apoptotic chromatin condensation inducer 1 (*ACIN1*) gene viewed on WashU Epigenome Browser. ChromHMM tracks for E062 primary mononuclear cells shown. Methylation CpG coverage displayed in blue lines.

**Supplementary** **Figure S3.** Validation of cg10846936 in blood from 407 COVID-19 participants obtained from GEO GSE168739.

**Supplementary** **Figure S4.** Epigenetic clock analyses of pre vs post-COVID-19 utilizing online DNA methylation age calculator.

**
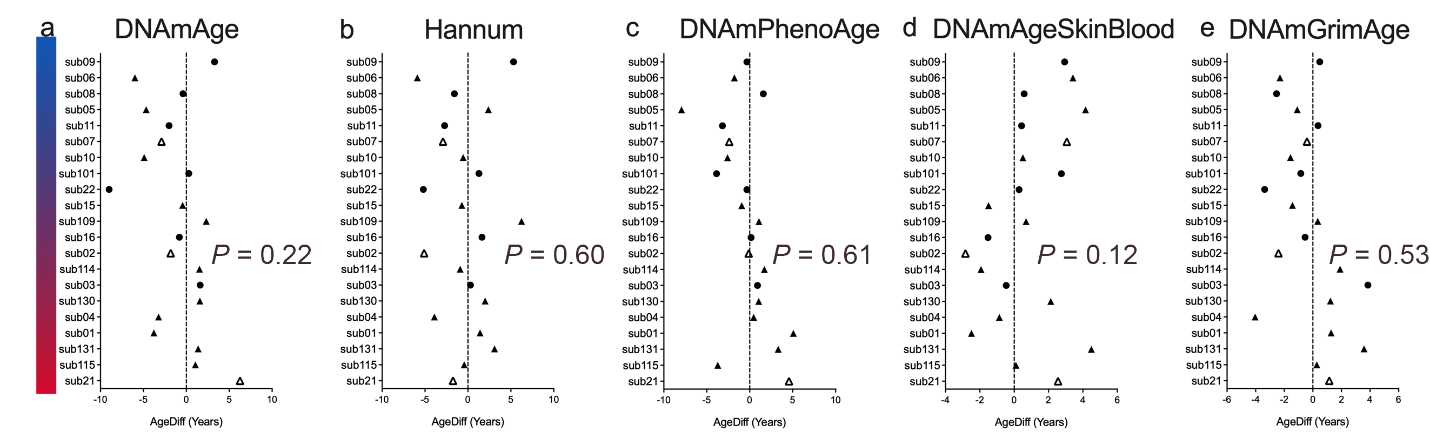
**

**Supplementary** **Figure S5.** DuneinPoAm45 and epiTOC analyses of pre vs post-COVID-19.


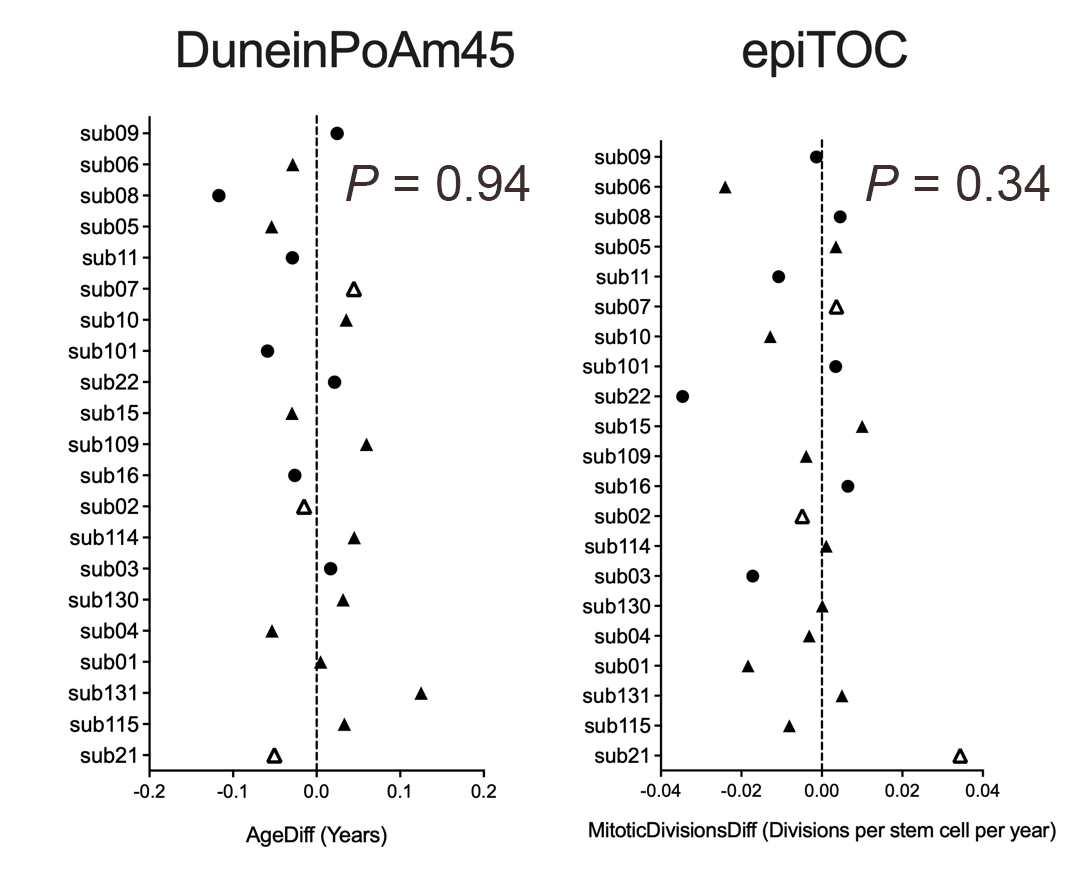


**Supplementary** **Figure S6.** Bar graph displaying mean +SD of time from last dose (days) for participants that received Moderna vaccine compared to Pfizer vaccine. Student’s t-test.

**Supplementary** **Figure S7.** PC-based epigenetic age estimates in Calu-3 and Human Donor PBMC exposed to SARS-CoV-2 *in vitro*.

**
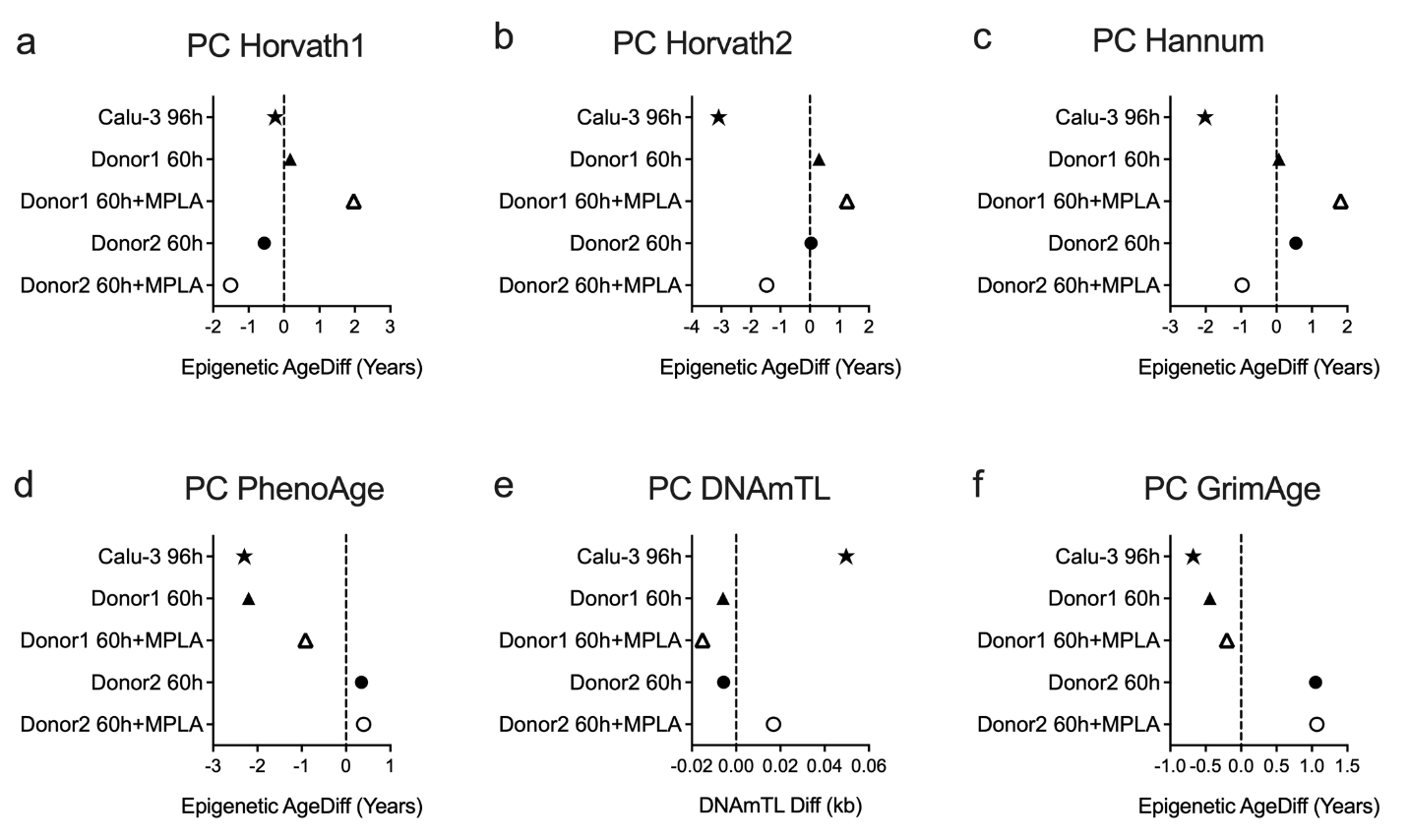
**

**Supplementary** **Figure S8.**

Validation of epigenetic age estimates in blood from participants at pre- and post-COVID-19 timepoints.
